# The architecture of sponge choanocyte chambers is well adapted to mechanical pumping functions

**DOI:** 10.1073/pnas.2421296122

**Published:** 2025-03-21

**Authors:** Takumi Ogawa, Shuji Koyama, Toshihiro Omori, Kenji Kikuchi, Hélène de Maleprade, Raymond E. Goldstein, Takuji Ishikawa

**Affiliations:** ^a^Department of Finemechanics, Tohoku University, Aramaki, Aoba-ku, Sendai 980-8579, Japan; ^b^Sorbonne Université, CNRS, Institut Jean Le Rond d’Alembert, Paris F-75005, France; ^c^Department of Applied Mathematics and Theoretical Physics, University of Cambridge, Cambridge CB3 0WA, United Kingdom; ^d^Department of Biomedical Engineering, Tohoku University, Aramaki, Aoba-ku, Sendai 980-8579, Japan

**Keywords:** Porifera, flagella, low Reynolds number flow, pumping

## Abstract

Sponges, the basalmost members of the animal kingdom, exhibit a range of complex architectures in which microfluidic channels connect multitudes of spherical chambers lined with choanocytes, flagellated filter-feeding cells. Choanocyte chambers can possess scores or even hundreds of such cells, which drive complex flows entering through porous walls and exiting into the sponge channels. One of the mysteries of the choanocyte chamber is its spherical shape, as it seems inappropriate for inducing directional transport since many choanocyte flagella beat in opposition to such a flow. Here, we combine direct imaging of choanocyte chambers in living sponges with computational studies of many-flagella models to understand the connection between chamber architecture and directional flow. We find that those flagella that beat against the flow play a key role in raising the pressure inside the choanocyte chamber, with the result that the flow rate and mechanical pumping efficiency reach a maximum at a small outlet opening angle. Comparison between experimental observations and the results of numerical simulations reveal that the chamber diameter, flagellar wave number, and the outlet opening angle of the freshwater sponge *Ephydatia muelleri*, as well as several other species, are related in a manner that maximizes the mechanical pumping functions. These results indicate the subtle balances at play during morphogenesis of choanocyte chambers, and give insights into the physiology and body design of sponges.

## Significance

Sponges are among the oldest multicellular animals. Their simple body plans make them a model organism for studies of both morphogenesis and physiology. Our study reveals that the choanocyte chamber diameter, flagellar wave number, and the outlet opening angle of the freshwater sponge *Ephydatia muelleri*, as well as several other species, are related in a manner that maximizes the mechanical pumping functions. These results indicate the subtle balances at play during morphogenesis of choanocyte chambers and give insights into the physiology and body design of sponges as well as their descendants, multicellular animals. The acquired knowledge is also of value in the design of an artificial ciliary pump.

**Fig. 1.**
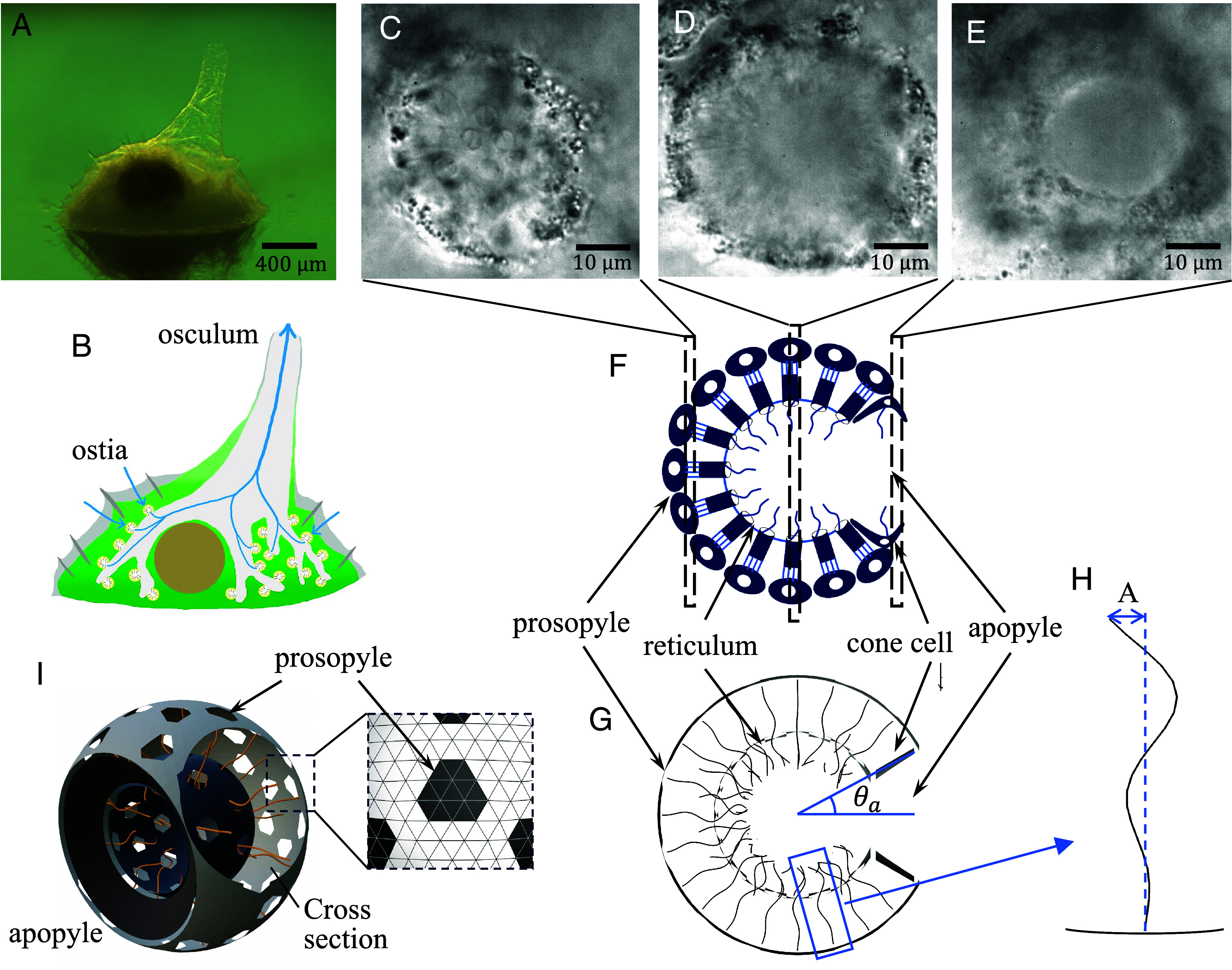
The fresh water sponge *E. muelleri* and computational model of choanocyte chamber. (*A*) Stereomicroscopic image, with the body shown reflected in glass. (*B*) Schematic diagram of the aquiferous canal system. Blue lines indicate the direction of water flow. (*C*–*E*) Microscopic images of the choanocyte chamber at the *Bottom*, *Middle*, and *Top* focal planes as indicated in the schematic (*F*). Parallel arrows indicate fluid flow into a choanocyte chamber, while blue shaded ellipse indicates location of the opening from which fluid exits. (*G*) Schematic cross-section of the model chamber. (*H*) Definition of the flagellar beat amplitude *A*. (*I*) Three-dimensional model of the choanocyte chamber.

Sponge-like fossils have been found that date as far back as 550 to 760 My (1). While the analysis of the evolution from unicellular to multicellular life remains unsettled (2–10), it remains likely that sponges are the oldest animals, and their simple body plans make them a model organism for studies of both morphogenesis and physiology. As filter feeders with a distinct pump-filter apparatus, sponges (phylum Porifera) can process several hundred times their body volume of water per hour (11, 12). Because of this, they play a significant role in nutrient cycling within marine ecosystems such as coral reefs (13–15). To achieve this high-performance filtering, sponges have an evolved aquiferous system that allows water to flow through their bodies (16, 17), and are divided into several classes (termed *asconoid*, *syconoid*, and *leuconoid*) according to the level of complexity of this internal microfluidic system (18). Leuconoid sponges have the most complex architecture, composed of incurrent and excurrent canals. Water entering their body through the inlets (ostia) on the surface of the sponge body is carried through the incurrent/excurrent canals and exits from the outlet (osculum), as shown in Fig. 1 for the case of the freshwater sponge *Ephydatia muelleri*. The spherical chambers act as pumps between the incurrent and excurrent canals and are lined with flagellated cells termed choanocytes arranged in a radial pattern with flagella directed toward the center of the sphere, supporting traveling waves of motion that direct flow toward the center. Filtering of incoming water is achieved by a collar of microvilli anchored near the apex from which emanates the flagellum of each cell.

The spherical arrangement of choanocytes (Fig. 1*C*–*F* appears at first glance to be ill-suited to yield directional flow through the chamber, since inevitably the flagella of some of the choanocytes would beat in opposition to the flow. Since sponges have survived from the distant past, it is natural to hypothesize that this spherical shape confers an evolutionary benefit. Here, we seek to understand this issue from the perspective of fluid mechanics.

Vogel’s pioneering early work was the first to highlight the fluid mechanics of sponges (19–21). While the properties of the “sponge pump” have been of interest since then (22, 23), they remain incompletely understood (24–26). One of the most important issues is the connection between large-scale flows external to the sponge and the flows within. Although the sizes of sponges span an enormous range, even taking a modest vertical scale $L∼10-10^{2}\;$cm and the typical ambient flow speeds $U∼10-10^{2}\;$cm/s the Reynolds number Re=UL/ν in water is 10^4^ to 10^5^ outside a sponge. With such a large *Re* we expect a viscous boundary to form at the sponge surface whose maximum thickness $\delta ∼x/\sqrt{Re_{x}}$, where *x* is distance along the sponge and $Re_{x}=Ux/\nu$. Using the representative x∼10cm we obtain δ∼1mm. Thus, the ostia through which water flows into the microfluidic network of the sponge sit within the viscous boundary layer. While the general problem of flow into a permeable wall has been studied in some detail (27–29), no connection has yet been made with sponge fluid dynamics.

The relationship between external and internal flows can then be recast in terms of the connection between Bernoulli pressure differences between the ostia and osculum set up by external flows and internal pressures created by choanocytes beating within the chambers. In this regard, there are conflicting conclusions in the literature. Dahihande and Thakur (30) reported that the number density of choanocyte chambers and the number of choanocytes per chamber are positively correlated with the pumping rate of sponges. On the other hand, Larsen and Riisgård (31) found that the pumping rate depends on the pressure losses of the aquiferous system with increasing sponge size and not on the reduced density of choanocytes.

Recently, there have been several important numerical studies of the sponge pump at the level of choanocytes. Asadzadeh et al. (32) found numerically that the both the glycocalyx mesh covering the upper part of the collar and secondary reticulum are important for the pump to deliver high pressure. They also reported that choanocytes arranged in a cylindrical configuration can pump water efficiently owing to the formation of a “hydrodynamic gasket” above the collars (33). This gasket is associated with lateral “vanes” which are known to exist on the basal part of the flagella inside the collar of certain species of sponges (34). It was hypothesized that in fitting tightly within the collar, the beating flagellum acts like a piston, displacing fluid with little leakage. This structure is difficult to preserve intact when preparing specimens for electron microscopy, and thus it is documented in only certain species. Notably, a recent study (35) finds that the vane width is at most half of the diameter of the collar, suggesting that the gasket effect would be significantly reduced from its hypothesized maximum.

Finally, we note that the spherical shape of choanocyte chambers has not discussed hydrodynamically in any of these former studies and the physiological significance of that shape is unclear. In recent years, ciliated pumps have gained considerable attention as a microscale fluidic transport method. Artificial ciliated platforms (36–38) and bacterial carpets (39) have been examined for their potential applications in microscale pumping and mixing processes. The majority of extant literature focuses on near-surface flow resulting from cilia arranged in a flat plane. However, this study offers a valuable opportunity to gain insight into the influence of the spherical configuration. Here, we examine the hydrodynamic properties of spherical choanocyte chambers by combining direct imaging of chambers in living sponges with computational studies of many-flagellum models of their fluid mechanics. We used freshwater sponges *Ephydatia muelleri* as a model organism for experimental observation and to define a computational model of the fluid mechanics of a choanocyte chamber.

Choanocyte chambers consist of choanocytes arranged in a spherical configuration, each with a single flagellum oriented toward the center of the chamber, propagating bending waves radially inward. In the computations, we represent the chamber as a rigid sphere of radius *R* from which flagella emerge, as in Fig. 1. The chamber has many small inlet holes termed *prosopyles* and one large outlet hole known as the *apopyle*. Prosopyle diameters vary in the range of 1 to 5 In our model, each prosopyle is represented by removing some mesh from spherical shell to satisfy $S_{p}/4\pi R^{2}∼10^{-3}$ (Fig. 1*I*). As many as possible were introduced without reducing the number density of flagella. Additionally, there is the gasket-like *reticulum*, a fine structure that connects the apical part of the collars, which was also observed in *Spongilla lacustris* (42). The reticulum delivers high pressure because the choanocyte acts as a collar-vane-flagellum pump system (32). However, the wing-like structure “vane” observed on flagella in several species of sponge (10, 35, 42, 43) were not seen in *E. muelleri* (Movie S1). The reticulum was modeled as a concentric spherical shell inside the choanocyte chamber (Fig. 1*G*), and its radius was varied with the chamber radius so that the length of the collar extending from the choanocyte was a constant 8.2 μm (10). Finally, *cone cells* are found in several freshwater sponges including *E. muelleri* (44) and are flattened near apopyles (42, 44, 45). They form a ring and connect collars with the walls of excurrent canals to prevent the disadvantageous current. The cone cell ring was modeled as a conical surface near the apopyle (Fig. 1*G*), with an opening angle *θ*_*a*_. The cone cell ring and reticulum play an important role in preventing backflow from the sides of the flagellum, which has the effect of increasing the flow rate and raising the pressure inside the choanocyte chamber. The geometric and dynamical characteristics of choanocyte chambers and the flagellar motion within them are listed in Table 1, in which the results of *S. lacustris* obtained by Mah et al. (10) are added for comparison.

**Table 1.** Characteristics of choanocyte chambers

Quantity	*E. muelleri*	*S. lacustris*	Simulation
Number of flagella *N*	112 ± 31		37 to 359
Flagellar length *L*	12.9 ± 1.1 μm	10.4 ± 0.3 μm	13 μm
Flagellar amplitude *A*	1.58 ± 0.21 μm	3.3 ± 0.9 μm	0.14 L
Beat frequency *ν*	26.1 ± 8.9 Hz	11.0 ± 1.1 Hz	
Chamber radius *R*	17.4 ± 2.5 μm		(1.5 to 3) L

Data for *S. lacustris* are from Mah et al. (10).

In this study, we show computationally that the flagella beating against the flow play a role in raising the pressure inside the choanocyte chamber and the mechanical pumping functions reaches a maximum at a certain outlet opening angle. Good agreement between the experimental and the numerical results, such as the flagella wave number and outlet opening angle, is presented in *Discussion*.

## Result

In describing the numerical computations, the various parameters of the setup can be recast in convenient dimensionless quantities. First, if *θ*_*a*_ is the opening angle of the apopyle, the area of the chamber available for choanocytes is $2\pi R^{2}\left(1+\cos\theta_{a}\right)$. We may view the flagellar undulation amplitude *A* (*SI Appendix*, Eq. **S9**) as defining an area $\pi A^{2}$ associated with each choanocyte, and thus it is natural to define the area fraction *ϕ* of choanocytes on the chamber surface as

[1] $$\begin{array}{c} \phi =\frac{NA^{2}}{2R^{2}\left(1+\cos\theta_{a}\right)}. \end{array}$$

For reference, in a hexagonal close packing on a flat surface, the maximum packing fraction is $\pi \sqrt{3}/6≃0.907$.

### Basic Observations.

**Fig. 2.**
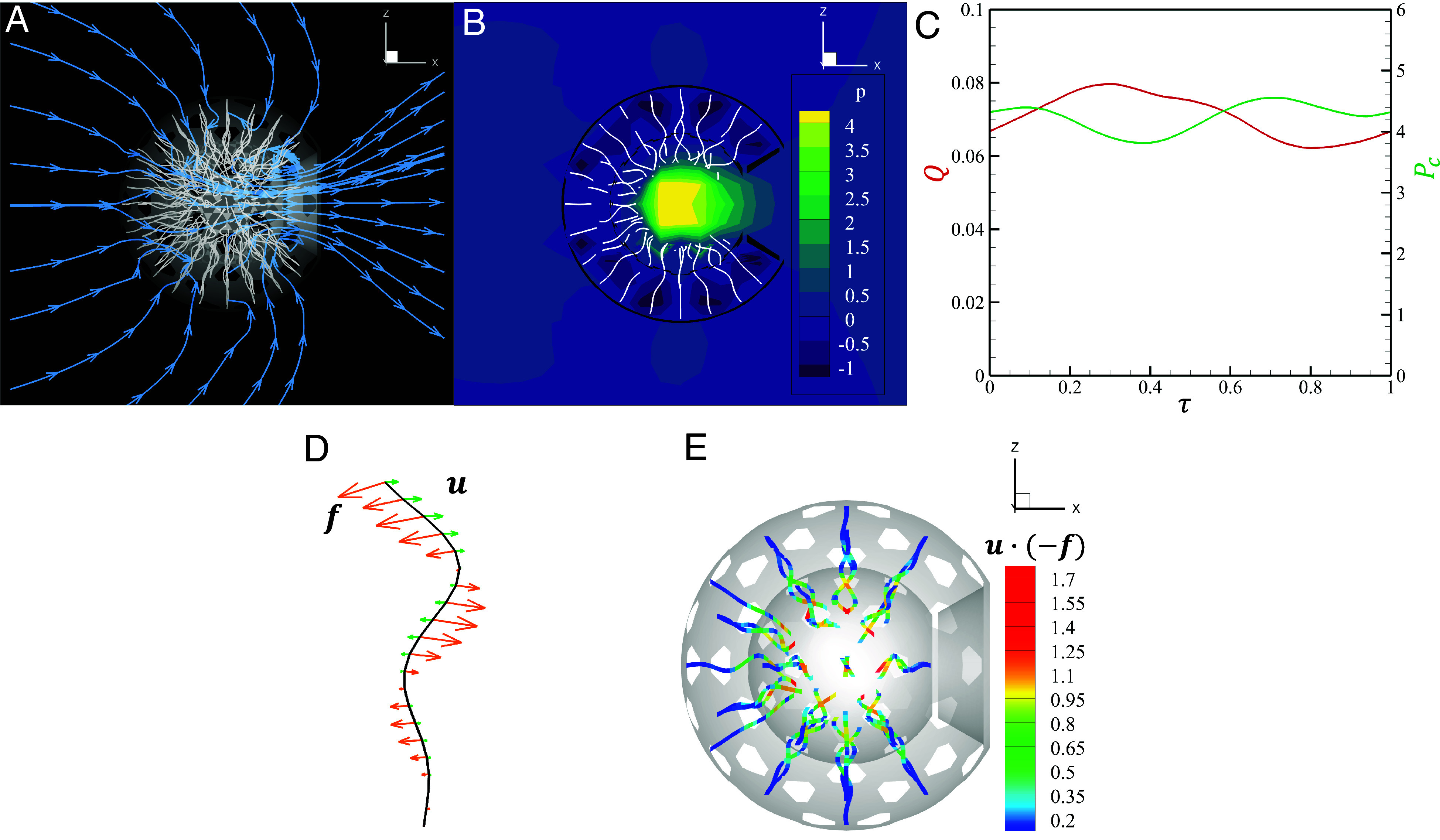
Numerical results. (*A*) Flow field around the model choanocyte chamber, with *ρ* = 1.5, *N* = 143 (*ϕ* = 0.33), $\theta_{a}=30^{{}^{\circ}}$, and kℓ=3π. The streamlines are drawn in the center cross-section. (*B*) Pressure distribution in the chamber. (*C*) Temporal behavior of the outlet flow rate and maximum pressure within the chamber. (*D*) Force density and velocity at node points of a flagellum. (*E*) Magnitude of the inner product of the force density and the velocity.

The time-averaged flow field and pressure in the center cross-section of a chamber are shown in Fig. 2 *A* and *B* for representative parameters. We confirmed that the unidirectional flow, from prosopyle to apopyle, is generated by flagellar beating in the spherical chamber. In this spherical geometry, the pressure field can be divided into low and high pressure regions, as previously reported (32, 42). The low pressure region sucks water from the prosopyle, while the high pressure region ejects water outward, leading to a unidirectional flow with volumetric rate $Q^{*}$ from the apopyle of

[2] $$\begin{array}{c} Q^{*}=\int u\cdot n\;dS_{a}, \end{array}$$

where *S*_*a*_ is the area of the curved surface of the apopyle, u is the velocity on *S*_*a*_, and n is the outward unit normal vector on *S*_*a*_. As the flagellar length is the fixed scale used for nondimensionalizing quantities, we use it and the beat period to define the rescaled flow rate

[3] $$\begin{array}{c} Q=\frac{Q^{*}T}{L^{3}} \end{array}$$

and its time-averaged value $\overset{¯}{Q}$. As shown in Fig. 2*C*, *Q* does not vary significantly with time. For the parameters *ρ* = 1.5, *N* = 143 (*ϕ* = 0.33), $\theta_{a}=30^{{}^{\circ}}$ and kℓ=3π we find $\overset{¯}{Q}=0.071$, a value that is compared with experimental results in *Discussion*. From the Stokesian balance $\nabla P^{*}=\mu \nabla^{2}u$, with u∼L/T, a suitable rescaled pressure is

[4] $$\begin{array}{c} P=\frac{P^{*}T}{\mu }, \end{array}$$

which is used to quantify the maximum pressure $P_{\max}$ and its time-average ${\overset{¯}{P}}_{\max}$. The time course of pressure at the center of chamber $P_{c}$ is shown in Fig. 2*C* because $P_{\max}$ appear around the center of chamber. The force density f and the velocity u of node points on a flagellum are shown in Fig. 2*D*. These can be used to determine the rate of working $W^{*}$ of the entire flagellum in moving fluid as

[5] $$\begin{array}{c} W^{*}=\sum_{i}^{N}\int u\cdot \left(-f\right)ds_{i}. \end{array}$$

With the force density f∼μu and speeds scaling as L/T, we define the dimensionless rate as

[6] $$\begin{array}{c} W=\frac{W^{*}T^{2}}{\mu L^{3}}. \end{array}$$

Intuitively, we see that the magnitude of u·(−f) of each flagellum is largest near the tip, as in Fig. 2*E*, which creates high pressure in the center of the chamber.

### Output Increase with Number of Flagella.

To clarify the relationship between the number of flagella in a chamber and its fluid dynamical properties, pumping functions were investigated as the number was varied from 10 to 347. The pumping function was evaluated according to the outlet flow rate *Q* from the apopyles, the rate of working of the flagella, the maximum pressure $P_{\text{max}}$ in the chamber and the mechanical pumping efficiency *η* averaged over time during a beat cycle of the flagella. The work done by the pump can be evaluated as the pressure rise multiplied by the volumetric flow. Here, we assume zero pressure at infinity, as the work of the pump alone is to be calculated. Then, *η* can be described as

[7] $$\begin{array}{c} \eta =\frac{\overset{¯}{Q}{\overset{¯}{P}}_{\text{max}}}{\overset{¯}{W}}, \end{array}$$

where overbars indicate time-averaged quantities. We note that the effect of the pressure difference between the inlets and outlet will be explicitly taken into account in *Discussion*.

**Fig. 3.**
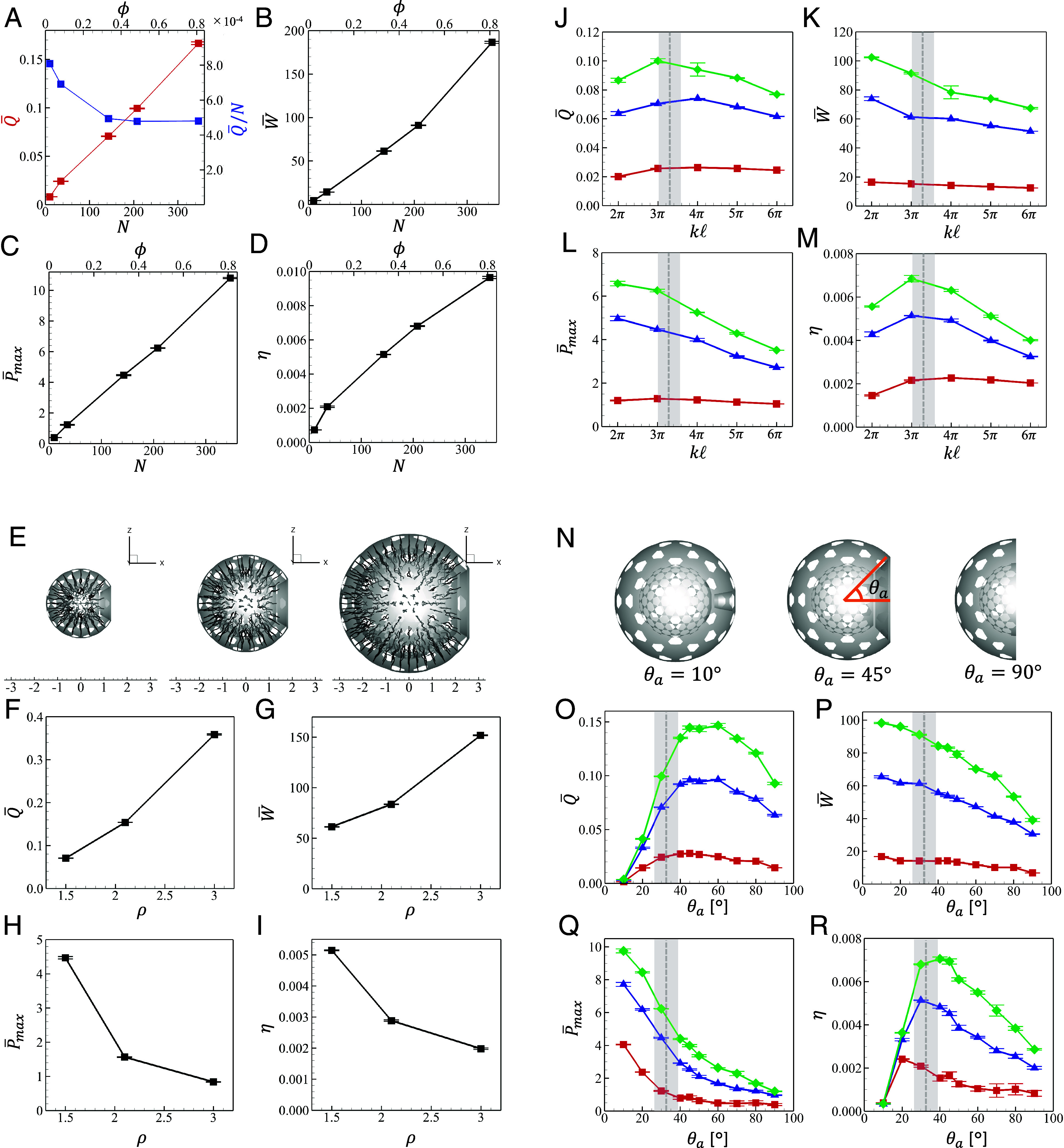
Numerical results. (*A*–*D*) Correlation of pumping functions with the number of flagella. (*A*) Outlet flow rate. (*B*) Work rate done by flagella. (*C*) Maximum pressure in the chamber. (*D*) Mechanical pumping efficiency. (*E*–*I*) Correlation of pumping function with chamber radius. The length and number density of flagella are fixed. (*E*) Computational domain for three values of *ρ*. (*F*) Outlet flow rate. (*G*) Work rate done by flagella. (*H*) Maximum pressure in the chamber. (*I*) Mechanical pumping efficiency. (*J*–*M*) Correlation between the pumping function and wave number of flagella, keeping area fraction *ϕ* in narrow ranges: red: 0.074 to 0.093, blue: 0.31 to 0.34, and green: 0.39 to 0.49. (*J*) Outlet flow rate. (*K*) Work rate done by flagella. (*L*) Maximum pressure in the chamber. (*M*) Mechanical pumping efficiency. (*N*–*R*) Correlation between pumping function and outlet opening angle. (*N*) Computational domain for three values of *θ*_*a*_. (*O*–*R*) Chamber properties as a function of opening angle, keeping area fraction *ϕ* in narrow ranges: red: 0.074 to 0.093, blue: 0.31 to 0.34, and green: 0.39 to 0.49. (*O*) Outlet flow rate. (*P*) Work rate of flagella. (*Q*) Maximum pressure in the chamber. (*R*) Mechanical pumping efficiency. Gray bars in (*J*–*M*) and (*O*–*R*) indicate the values of experimental observations.

In Fig. 3*A*–*D*, ensemble averaged $\overset{¯}{Q}$, $\overset{¯}{W}$, ${\overset{¯}{P}}_{\text{max}}$, and *η* are shown with three independent phases and rotation angles of the flagellar beating planes. The larger the number of flagella (and the greater the packing fraction *ϕ*), the greater $\overset{¯}{Q}$, $\overset{¯}{W}$, ${\overset{¯}{P}}_{\text{max}}$, and *η*, indicating better performance as a pump. Most notably, the average flux $\overset{¯}{Q}$ is very nearly linear in *N* and the flux per flagellum $\overset{¯}{Q}/N$ saturates to a constant value of $5\times 10^{-4}$ at large *N*. In interpreting this value it is useful to adopt a simplified view of the flagellum as a localized point force acting on the fluid, a model that has experimental support from studies of *Chlamydomonas reinhardtii* (46). The associated Stokeslet flow field, if examined in free space without boundaries, has an infinite flux through any plane orthogonal to the direction of the point force, while for a force orthogonal to a nearby no-slip surface the flux vanishes by fluid conservation. This latter result arises from compensating flows away from the surface near the singularity and toward the surface far away from it. A numerical computation of the flux associated only with the outgoing flows driven by a single model flagellum used in the present calculations, attached to a no-slip wall, gives a value of ∼0.01, a factor of 20 greater than the limit seen in Fig. 3*A*. While the porosity of the wall would tend to increase the flux, the tight confinement within the chamber and the typically small apopyle angle clearly leads to significant cancellation. For a given chamber radius, excluded volume effects from cells and their collars limits the number of choanocytes that can be spherically aligned, a limit that depends on the size and shape of the choanocyte. The choanocyte diameter varies among species: 3μm for *Tetilla serica* (47), 3.7 to 5 μm with pseudocylindrical shape for *Halisarca dujardini* (48), 6μm for *E. fluviatilis* (49), and 5μm for *E. muelleri* (50).

### A Smaller Chamber Radius Increases Efficiency.

We investigated the effect of the chamber radius on the pumping functions such as the efficiency by varying the radius from 1.5 L to 3.0 L while keeping the number density of flagella fixed. As shown in Fig. 3*F*–*I*, the flow rate and work rate increased with the chamber radius as the number of flagella also increased. On the other hand, the maximum pressure decreased as the chamber radius increased. The pressure drop was due to the large center space within the chamber, where there are no direct flagella forces. As a result, the mechanical pumping efficiency decreased with increasing radius. These results indicate that a larger chamber has no efficiency advantage, but rather that it is advantageous to pack more choanocytes into a smaller chamber.

### Flow Rate and Efficiency Is Maximized at Intermediate *kℓ*.

We studied the effect of changing the flagellar wave number while keeping the flagellar length fixed. Interestingly, as shown in Fig. 3*J*–*M*, the outlet flow rate and mechanical pumping efficiency exhibit peaks at intermediate values of *k*, while the particular value of the peak *k* differs between the two. The mechanical pumping efficiency reaches a maximum when at the relatively low wave number kℓ=3π, where *ℓ* is projected flagellar length. That higher wave numbers lead to reduced efficiency, despite the reduced work rate of the flagella, arises from an effect similar to that found when the chamber radius is reduced; Since *L* is fixed, *ℓ* shrinks at higher wave numbers, increasing the space at the chamber center from which flagella are absent, and the central pressure reduces with higher wave number. The wave number that maximizes the efficiency is compared with the experimental results in *Discussion*.

### Flow Rate and Efficiency Is Maximized at Intermediate *θ*_*a*_.

While the choanocyte chamber diameter, apopyle area, and diameter were examined in previous studies (22, 40, 51), it has remained unclear how the apopyle’s aperture ratio affects the pumping function of the chamber. We examined the pumping function $\theta_{a}\in \left[10^{{}^{\circ}},90^{{}^{\circ}}\right]$ while keeping the area fraction *ϕ* within each of three narrow ranges, adjusting the number of flagella with *θ*_*a*_ accordingly. Fig. 3*O*–*R* shows the variation in pumping functions with *θ*_*a*_. With all three flagella densities, similar trends were observed, with small variations in the position of various peaks. The outlet flow rates reaches a maximum at $\theta_{a}∼$ 40^°^ to 60^°^. On the other hand, the maximum pressure decreases monotonically with *θ*_*a*_, an effect that arises from the reduction in the number of flagella directed against the bulk flow as *θ*_*a*_ increases. Thus, the spherical shape of the choanocyte chamber has the effect of increasing pressure. The same conclusion has been reached in a coarse-grained point force model of the choanocyte chamber (cf. *SI Appendix*, section 3). Since the pumping efficiency is the product of the maximum pressure and the flow rate, its peak in Fig. 3*R* shifts to the lower *θ*_*a*_ regime of $20^{{}^{\circ}}$ to $50^{{}^{\circ}}$ compared to the case of the flow rate (cf. Fig. 3*O*). From these results, we conclude that flagella around apopyles, which seem to disturb unidirectional flow, contribute to creating the high pressure rise and that the choanocytes with intermediate but small *θ*_*a*_ can achieve high mechanical pumping efficiency.

## Discussion

### Pressure Difference Across the Chamber.

**Fig. 4.**
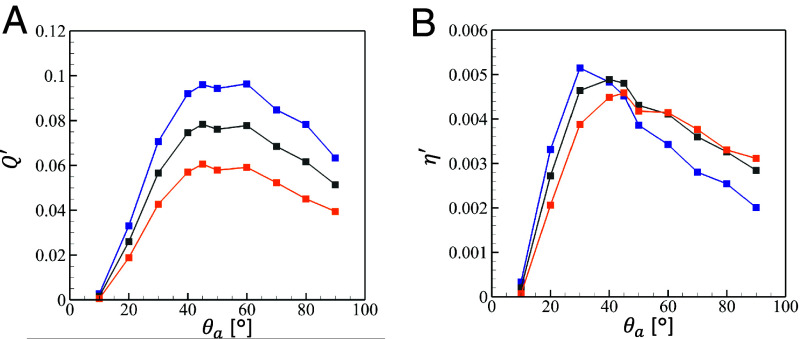
Effect of pressure difference ΔP at the case of *ϕ* = 0.31 to 0.34. (*A*) the pumping flow rate and (*B*) the mechanical pumping efficiency: blue: ΔP=0 (0 Pa), gray: ΔP=1 (0.026 Pa), and orange: ΔP=2 (0.052 Pa).

In the above, the choanocyte chamber model was set in a context devoid of opposing pressure. When the pressure at the apopyle is higher than that at the prosopyles, the pumping flow rate and the pressure in the chamber are affected by the pressure difference ΔP. Here, we discuss the effect of ΔP under the assumption that the flagellar beat pattern is unaffected by ΔP. By exploiting the superposition of the Stokes flow solutions, the setting of the beating flagella under ΔP can be split into two subsettings; a) beating flagella without ΔP, and b) immotile flagella with ΔP. The pumping flow rate and maximum pressure can then be estimated as $Q^{\prime }=\overset{¯}{Q}+C_{1}\Delta P$, $P^{\prime }={\overset{¯}{P}}_{\;\text{max}}+C_{2}\Delta P$. The method for determining the value of *C*_1_ and *C*_2_ is detailed in *SI Appendix*, section 4. The mechanical pumping efficiency $\eta^{\prime }$ is calculated by applying $Q^{\prime }$ and $P^{\prime }$ to Eq. **7**. As shown in Fig. 4*A*, $Q^{\prime }$ shift to decrease for increasing ΔP. In Fig. 4*B*, there is a slight shift in the peaks of $\eta^{\prime }$, however, the underlying trend remains consistent.

### Pumping Flow Rate.

The numerical results, with parameters taken from experiment, yielded time-averaged flow rates of a choanocyte chamber of $\overset{¯}{Q}≃4\times 10^{3}\mu {\text{m}}^{3}/s$. This can be compared with previous experimental results. The flow rate of the choanocyte chamber of *Haliclona urceolus* was estimated as $Q=4.5\times 10^{3}\mu {\text{m}}^{3}/s$ (22), which is very close to the present results. In addition, the flow rate per choanocyte of a syconoid type sponge was estimated at 48μm^3^/s (33), and if the flow rate scales with the number of choanocytes, estimated to be 100 (cf. Table 1), we obtain $Q=4.8\times 10^{3}\mu$m^3^/s, also in good agreement with our results. Thus, the flow rate obtained in this study is consistent with former experimental observations.

### Flagellar Wave Number.

**Fig. 5.**
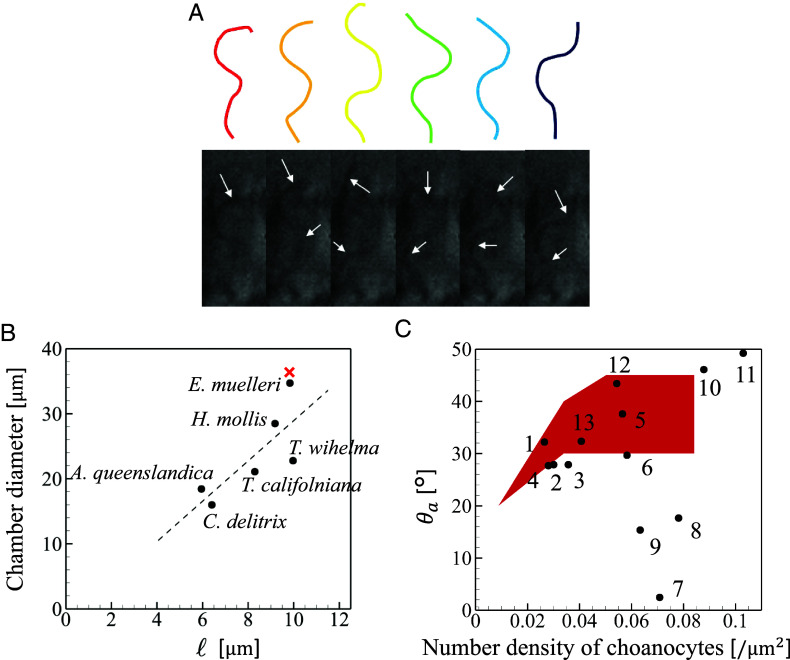
(*A*) Flagellar motion of a choanocyte of *E. muelleri*. White arrows indicate flagella. Colored curves represent extracted flagellar waveforms. (*B*) Correlation between the projected flagellar length and the choanocyte chamber diameter. If not specified, the values were measured manually from images; *Tethya wilhelma* from Hammel et al. (25); *Haliclona mollis*, *Tethya californiana*, and *Cliona delitrix* from Ludeman et al. (24); *Amphimedon queenslandica* from Sogabe et al. (52). × indicates the hypothetical minimum diameter of *E. muelleri* that is obtained under the condition that adjacent flagella do not touch. (*C*) Correlation of the opening angles of apopyles and the number density of choanocyte. Black symbols and numbers correspond to the species listed in Table 2. The red area indicates the angle at which the numerically obtained mechanical pumping efficiency reaches greater than 0.9 times its maximum value at each of four number densities used in the simulation.

Next, we compare the wave number at which the efficiency and flow rate is maximized with our experimental results. The mechanical pumping function of the wave number with three different number densities of flagella is shown in Fig. 3*J*–*M*. We see peaks in efficiency and flow rate with the kℓ∼(3to4)π, independent of the number density of flagella. In our experiment, the flagellar motion of *E. muelleri* choanocyte was extracted from the high-speed imaging as shown in Fig. 5*A*. From the projected flagellar length ℓ=9.83±0.74μm and the wavelength λ=5.98±0.48μm, we find kℓ=(3.30±0.27)π, in good agreement with the numerical results for the maximum efficiency and flow rate (Fig. 3*M*). Similar trends are seen in other species. In the case of *S. lacustris*, for example, kℓ∼4π using ℓ=10.4μm and λ=5μm (10, 32). Thus, the flagellar wave number appears to be tuned to maximize the mechanical pumping efficiency and outlet flow rate.

### Chamber Size and Choanocyte Density.

Our experimental observations show that the number of flagella in a choanocyte chamber of *E. muelleri* is ∼100, similar to the value ∼80 found in earlier work on choanocytes of *H. urceolus*, in which the chamber diameter is ∼30 μm (22). In several marine sponges, the chamber diameters range from 16 to 31 μm and the number of choanocytes ranges from 32 to 130, which indicates that choanocytes are densely packed in the chamber. There is a positive correlation between the number of choanocytes per chamber and the pumping rate of a sponge according to experimental observations (30). Hence, it is inferred that the chamber is filled almost completely with as many choanocytes as allowed by the chamber size. These tendencies are consistent with our finding that larger number of flagella enhances pumping function and efficiency.

As noted earlier, there is an upper limit on the number density of choanocytes due to the excluded volume of cells and the aperture ratio of apopyles and prosopyles. Hence, to increase the number of choanocytes, the choanocyte chamber is forced to make its radius larger. On the other hand, when the radius is large, the pumping efficiency becomes low. These two conditions are contradictory, so a balance must be reached between them. To find balanced conditions in nature, we plotted the correlation of the flagellar length and the choanocyte chamber diameter for various species of sponge as shown in Fig. 5*B*. We see an obvious positive correlation between them, indicating that the chamber diameter increases with flagellar length. We analytically calculated the hypothetical minimum diameter of *E. muelleri* under the condition that adjacent flagella of amplitude 1.58 μm do not contact each other. The result is 36.4 μm, which is close to the actual diameter of 34.7 μm. This implies that the chamber diameter is designed to be as small as possible while avoiding overlapping flagella. Smaller chamber diameters may have other advantages, such as the ability to accommodate a larger number of cells in a smaller volume and greater flexibility in the design of the canal in the entire of sponge body.

### Outlet Opening Angle.

Finally, we compare the experimentally observed opening angles with the range $\theta_{a}=20^{{}^{\circ}}\;\;\text{to}\;50^{{}^{\circ}}$ found to be maximal for pumping efficiency and be large flow rate. We observed several cross-sections of a choanocyte chamber of *E. muelleri* by shifting the focal plane as shown in Fig. 1*C*–*F*. From the image around the apopyle (cf. the *Top* focal plane in Fig. 1*E*), we measure the apopyle diameter. Using this value and the chamber diameter, and assuming a spherical chamber shape, the average apopyle opening angle was found to be $\theta_{a}=32.2\pm 6.7^{{}^{\circ}}$ (Table 2). In the case of *E. muelleri*, the number density of flagella is $0.032/\mu m^{2}$, which, with the chamber diameter of 34.7μm, and the flagella number of 112 gives a coverage fraction *ϕ* = 0.25, associated with the blue data in Fig. 3*R* for pumping efficiency, which is maximized at $\theta_{a}=30^{{}^{\circ}}\;\;\text{to}\;45^{{}^{\circ}}$. The very good agreement with the observed value for *E. muelleri* indicates that the natural configuration of the choanocyte chamber of *E. muelleri* optimizes the mechanical pumping functions.

**Table 2.** Dimensions of choanocyte chambers

	Chamber diameter (μm)	Apopyle diameter (μm)	Apopyle area (μm^2^)	*θ*_*a*_ (^°^)	Choanocytes per chamber
1. *Ephydatia muelleri*	34.7 ± 5.0	18.1 ± 4.3		32.2 ± 6.7	112 ± 31
2. *Haliclona urceolus*	30	14		27.8	80
3. *Haliclona permollis*	30	14		27.8	95
4. *Aphrocallistes vastus*	56	26		27.7	260
5. *Neopetrosia problematica*	23.3	16.0		43.4	80
6. *Haliclona mollis*	28.5	14.1		29.7	139
7. *Tethya californiana*	21.1	0.90		2.44	99
8. *Callyspongia vaginalis*	19.7	5.97		17.6	93
9. *Cliona delitrix*	16.0	4.23		15.3	50
10. *Amorphinopsis foetida*	17.46 ± 0.13		124.11	46.1	84.11 ± 3.02
11. *Callyspongia* sp.	19.35 ± 0.2		168.54	49.2	121.11 ± 4.33
12. *Haliclona* sp.	19.69 ± 0.23		113.21	37.6	68.83 ± 2.82
13. *Ircinia fusca*	30.68 ± 0.29		211.25	32.3	120.35 ± 8.98

1: Observations of 5 different chambers in 3 different sponges. 2: Data from Larsen et al. (22). 3 to 9: Data from Ludeman et al. (24). 10 to 13: Data from Dahihande et al. (30).

The geometric properties of choanocyte chambers in many sponge species that have been reported previously are collected in Table 2. To compare the present study with these former ones, we plotted the opening angle *θ*_*a*_ versus the number density of choanocytes in a chamber. In estimating these values, we assumed the chamber is spherical. The results are plotted in Fig. 5*C*, in which the present results on those values of *θ*_*a*_ that correspond to the maximum mechanical pumping efficiency at various densities are indicated in red. The opening angles of natural sponge choanocyte chambers are clearly close to the computational results for those with maximum efficiency, thus demonstrating that many species of sponge have choanocyte chambers that, like *E. muelleri*, can achieve high mechanical pumping efficiency.

## Conclusion

In this study, we combined direct imaging of choanocyte chambers in living sponges with computational studies of many-flagellum models of their fluid mechanics to unravel the biological significance of the spherical shape of choanocyte chambers. We determined that there are ideal conditions for achieving maximum mechanical pumping efficiency and pumping flow rate for such a geometry. In addition, our computational analysis revealed that those flagella that beat against the dominant flow play a role in raising the pressure inside the choanocyte chamber. As a result, the mechanical pumping efficiency—calculated from the pressure rise and flow rate—reaches a maximum at a modest outlet opening angle. Given that high pumping efficiency is achieved in many species (cf. Fig. 5*C*), the choanocyte chamber may have evolved to optimize this feature, which can be viewed as a means of overcoming the high fluid dynamical resistance of the complex canal.

## Materials and Methods

We used fresh water sponge *E. muelleri* for experimental observation. Details of harvesting, culturing, and imaging are described in *SI Appendix*, section 1.

In the numerical simulations for low Reynolds number flow, we solve the Stokes equation using the boundary element method with slender-body theory. Details of numerical methods are also in *SI Appendix*, section 2. To investigate the effects of the choanocyte chamber geometry on the pumping function of the chamber, in the numerical studies reported above we studied a range of several parameters of the computational modes: number of flagella N∈[37to359], chamber radius R∈[1.5L,3L] (with L=13μm), scaled wavenumber kℓ∈[1.5π,6π], and apopyle opening angle $\theta_{a}\in \left[10^{{}^{\circ}},90^{{}^{\circ}}\right]$. Through all computations, the calculational time step Δt was set to 0.02T, where *T* is the flagellar beat period; we also used this in the time averaging method.

## Supplementary Material

Appendix 01 (PDF)

Movie S1. Sectional observation of a choanocyte chamber of *E. muelleri*. This movie speed was decreased 0.25-fold.

## Data Availability

All data and analysis software are available on Zenodo (53).
