# Supplementary material for: The architecture of sponge choanocyte chambers is well adapted to mechanical pumping functions
Source: Proc Natl Acad Sci U S A. 2025 Mar 21;122(12):e2421296122. doi: 10.1073/pnas.2421296122 (PMC11962469; doi:10.1073/pnas.2421296122)
Supplement: Supplementary file 1 — Appendix 01 (PDF) [file pnas.2421296122.sapp.pdf]

PNAS

Supporting Information for

The Architecture of Sponge Choanocyte Chambers Maximizes Mechanical Pumping Efficiency

Takumi Ogawa, Shuji Koyama, Toshihiro Omori, Kenji Kikuchi, Hélène de Maleprade, Raymond E. Goldstein, and Takuji Ishikawa

Takumi Ogawa
E-mail: takumi.ogawa.t2@dc.tohoku.ac.jp

This PDF file includes:

Supporting text
Figs. S1 to S4
Legend for Movie S1
SI References

Other supporting materials for this manuscript include the following:

Movie S1

Supporting Information Text

1. Experimental methods

Harvesting and culturing of sponges: Freshwater sponges of the species *Ephydatia muelleri*, shown in Fig.1(a), living on natural stones were collected from the Hirose River (Izumi, Sendai City, Miyagi, Japan). The asexually produced buds of reproductive cells known as gemmules were peeled off, and the spicules on them were removed. To remove impurities, the gemmules were washed with a 1% aqueous solution of H_2O_2, followed by three successive rinses with pure water to remove excess H_2O_2. The purified gemmules were stored in a refrigerator at 4°C. Sponges were cultured in a plastic dish with Strekal's medium at room temperature (25°C).

Imaging: Choanocyte chambers of *E. muelleri* were imaged on an inverted microscope (IX71, Olympus Corp., Tokyo, Japan), as shown in Fig. S1(a). Flagellar beating in the chambers was imaged with a oil immersion objective (100×, N.A.= 1.40) and a high speed camera (500 fps, 1024×1024 pixels, FASTCAM SA3, Photron Limited, Tokyo, Japan) for durations of 5 s. The beating appears as a spatiotemporal brightness fluctuation in the pixels of the images (1, 2), as shown in Fig. S1(b), from which the beat frequency was measured from a fast Fourier transform (n=5, N=3: this means 5 different choanocyte chambers in 3 different sponges). The various geometric and dynamical characteristics of choanocyte chambers (n=6-8, N=4) and the flagellar motion within them (n=3-5, N=4) are shown in Table 1.

2. Computational fluid dynamics of choanocyte chambers

Governing equations: Scaled by the flagellar motion, the Reynolds number $Re \ll 1$ in a chamber, so the fluid flow in and around choanocyte chambers is governed by the Stokes equations. In the context of the situation in which a point force is acting at position $\boldsymbol{r}$ in incompressible Newtonian fluid, the Stokes equations with the forcing term take the form

$$\mu\nabla^2\boldsymbol{v} - \boldsymbol{\nabla}p + \boldsymbol{F}\cdot\delta(\boldsymbol{r}) = 0, \qquad [1]$$

where μ is the viscosity, $\boldsymbol{v}$ is the velocity, p is the pressure, $\boldsymbol{F}$ is the force, and $\delta(\boldsymbol{r})$ is the Dirac delta function. The fundamental solution of Eq. 1 in free space is

$$\boldsymbol{v} = \frac{1}{8\pi\mu}\boldsymbol{J}(\boldsymbol{r})\cdot\boldsymbol{F}, \qquad [2]$$

where $\boldsymbol{J}$ is the Green's function, also called the *Stokeslet* (3). It is given by

$$J_{ij}(\boldsymbol{x},\boldsymbol{y}) = \frac{\delta_{ij}}{r} + \frac{r_i r_j}{r^3}, \qquad [3]$$

where $r = |\boldsymbol{r}|$ and $\boldsymbol{r} = \boldsymbol{x} - \boldsymbol{y}$. When we consider the viscous traction $\boldsymbol{q}$ on the surface of a rigid body, Eq. 2 can be rewritten as a boundary integral equation (3),

$$\boldsymbol{v}(\boldsymbol{x}) = \frac{1}{8\pi\mu}\int \boldsymbol{J}(\boldsymbol{x},\boldsymbol{y})\cdot\boldsymbol{q}(\boldsymbol{y})dS(\boldsymbol{y}). \qquad [4]$$

Flagella have very slender structure with a sufficiently small diameter compared to the length. Consequently, the flagellum can be conceptualized as a curved filament, and described by slender-body theory (4).

Their centerlines are parameterized by arclength $s \in [0, L]$, and we measure the chamber radius R in units of the flagellar length, with

$$\rho = \frac{R}{L} \qquad [5]$$

a parameter that controls the size of the central region of the chamber devoid of flagella. The velocity $\boldsymbol{v}$ at point $\boldsymbol{x} \in s_i$ located on flagellum i can be written as (5)

$$\boldsymbol{v}(\boldsymbol{x}) = -\frac{1}{8\pi\mu}\int_{ch}\boldsymbol{J}(\boldsymbol{x},\boldsymbol{y})\cdot\boldsymbol{q}(\boldsymbol{y})dS(\boldsymbol{y}) - \frac{1}{8\pi\mu}\sum_{j}^{N}\int_{fla}\boldsymbol{K}(\boldsymbol{x},\boldsymbol{y})\cdot\boldsymbol{f}(\boldsymbol{y})ds_j(\boldsymbol{y}), \qquad [6]$$

where $\boldsymbol{f}$ is the force density of the flagella and N is the total number of flagella. The first integral is over all surfaces S, including those of the chamber, reticulum and cone cell ring. The second integral is over flagellar centerlines. In Eq. (6), $\boldsymbol{K}$ is the kernel (4)

$$K_{ij} = \frac{\delta_{ij}}{b} + \frac{r_i r_j}{b^3} + \frac{\varepsilon^2 L^2 a^2(s')}{2}\left(\frac{\delta_{ij}}{b^3} - \frac{r_i r_j}{b^5}\right), \qquad [7]$$

where $b = \sqrt{r^2 + \varepsilon^2 a^2(s)}$. Here the radius function a satisfies $0 < a(s) \le 1$ for each $s \in [0, \sqrt{L^2+\varepsilon^2}]$, where ε is the ratio of the length to the radius of flagellum and is set to $\varepsilon = 0.01$. The radius function $a(s)$ is

$$a(s) = \frac{1}{\sqrt{L^2+\varepsilon^2}}\sqrt{L^2+\varepsilon^2 - s^2}. \qquad [8]$$

When the point $\boldsymbol{x}$ is not on a flagellum, the velocity is

$$\boldsymbol{v}(\boldsymbol{x}) = -\frac{1}{8\pi\mu}\int_{ch} \boldsymbol{J}(\boldsymbol{x},\boldsymbol{y})\cdot\boldsymbol{q}(\boldsymbol{y})dS(\boldsymbol{y}) - \frac{1}{8\pi\mu}\sum_{j}^{N}\int_{fla}\left[\boldsymbol{J}(\boldsymbol{x},\boldsymbol{y})+\boldsymbol{V}(\boldsymbol{x},\boldsymbol{y})\right]\cdot\boldsymbol{f}(\boldsymbol{y})ds_j(\boldsymbol{y}), \qquad [9]$$

where (4, 6)

$$V_{ij} = \frac{\varepsilon^2 L^2 a^2(s')}{2}\left(\frac{\delta_{ij}}{r^3} - \frac{r_i r_j}{r^5}\right). \qquad [10]$$

The pressure at point $\boldsymbol{x}$ is (3, 7)

$$\begin{aligned} P^*(\boldsymbol{x}) = &-\frac{1}{8\pi\mu}\int_{ch}\boldsymbol{p}(\boldsymbol{x},\boldsymbol{y})\cdot\boldsymbol{q}(\boldsymbol{y})dS(\boldsymbol{y}) \\ &-\frac{1}{8\pi\mu}\sum_{i}^{N}\int_{fla}\boldsymbol{p}(\boldsymbol{x},\boldsymbol{y})\cdot\boldsymbol{f}(\boldsymbol{y})ds_i(\boldsymbol{y}), \end{aligned} \qquad [11]$$

where $\boldsymbol{p} = 2\boldsymbol{r}/r^3$. Contribution of the slender kernel to the pressure field is neglected as it decays quickly.

Flagellar motions: Each of the flagella within a computational choanocyte chamber is actuated with a prescribed waveform at a frequency $\nu = 1/T$, where T is the period of motion, and fixed at its base on the choanocyte chamber surface at a point $\boldsymbol{x}_b$. We define the orthonormal body frame $\boldsymbol{g}_1$ and $\boldsymbol{g}_2$ at $\boldsymbol{x}_b$ as $\boldsymbol{g}_1(\boldsymbol{x}_b) = -\boldsymbol{n}(\boldsymbol{x}_b)$, and $\boldsymbol{g}_2(\boldsymbol{x}_b) = \boldsymbol{b}(\boldsymbol{x}_b)\wedge\boldsymbol{n}(\boldsymbol{x}_b)/|\boldsymbol{b}(\boldsymbol{x}_b)\wedge\boldsymbol{n}(\boldsymbol{x}_b)|$, where $\boldsymbol{b}(\boldsymbol{x}_b) = \boldsymbol{e}_1\wedge\boldsymbol{n}(\boldsymbol{x}_b)$, $\boldsymbol{e}_1 = (1,0,0)$ and $\boldsymbol{n}(\boldsymbol{x}_b)$ is the outward unit normal vector to the surface. With $\tau = t/T$, the motion of a flagellum is parameterized as

$$\boldsymbol{x}^{fla} = \xi\boldsymbol{g}_1(\boldsymbol{x}_b) + \frac{A\xi}{\ell(t)}\cos\left(\frac{kL\xi}{\ell(t)} - 2\pi\tau + \varphi\right)\boldsymbol{g}_2(\boldsymbol{x}_b), \qquad [12]$$

where A is the beat amplitude, $k = 2\pi/\lambda$, with λ the wavelength, the coordinate $\xi\in[0,\ell(t)]$ spans the time-dependent *projected* length $\ell(t)$ of the flagellum under the constraint of fixed total arclength L. For the oscillating flagellum described by Eq. (12), the projected arclength ℓ is considerably less than the total arclength L. For the amplitude $A = 0.14L$ used in numerics and for the values of $k\ell\sim(3-4)\pi$ typical of experiment we have $\ell/L\sim 0.76$. This contraction plays an important role in the pressure distribution within the choanocyte chamber. As we observed no phase synchrony in our studies of sponge flagella, much as earlier studies of multicellular choanoflagellates saw no synchrony (8, 9), in computations we randomly set the phase φ for each flagellum in the range $\varphi\in[0,2\pi]$, reproduce the independent flagellar motion in the chamber.

Boundary element method: When the choanocyte chamber is fixed and there is no background flow, the boundary conditions are

$$\boldsymbol{v}(\boldsymbol{x}) = \begin{cases} 0 & \boldsymbol{x}\in\text{chamber} \\ \boldsymbol{v}^{fla}(\boldsymbol{x}) & \boldsymbol{x}\in\text{flagella} \end{cases} \qquad [13]$$

where $\boldsymbol{v}^{fla} = \partial\boldsymbol{x}^{fla}/\partial t$ is the flagella velocity, and the chamber includes the reticulum and cone cell ring.

The choanocyte chamber surface, reticulum and cone cell ring were composed of 9,710 triangular mesh elements and 5,401 nodal points. These quantities depended on the chamber radius, the size of apopyles, and the number of flagella. The prosopyles and apopyle were represented by removing twelve meshes from a rigid sphere discretized by a triangular mesh, as shown in Fig. S2(b). The remaining portion is treated as a wall to which the no-slip condition was applied as in Eq. (13). The flow rate through the prosopyle was calculated using Gaussian integration at the removed meshes.

To check the mesh convergence, we have generated the finest mesh currently available in our model, where the rigid sphere consists of 20,480 meshes and 10,242 nodes, as shown in Fig. S2(a). For each prosopyle, 52 meshes were removed (black area of Fig. S2(a)). The flow rates through the apopyle and the prosopyles are shown in Fig. S2(c). The difference between the flow rates obtained by the fine mesh and the coarse mesh is less than 8%. Given the small error and the reduced computational cost, the model depicted in Fig. S2(b) was adopted.

Each flagellum was discretized into 20 elements with 21 nodes. All physical quantities were computed at each discretized point. The boundary integral equations Eq. (6) and Eq. (9) were computed using Gaussian integration, leading to the linear algebraic equations

$$[\boldsymbol{v}^c] = [J^{cc}][\boldsymbol{q}] + [J^{cf}][\boldsymbol{f}], \qquad [14]$$

$$[\boldsymbol{v}^f] = [J^{fc}][\boldsymbol{q}] + [J^{ff}][\boldsymbol{f}]. \qquad [15]$$

Both $[\boldsymbol{v}^c]$ and $[\boldsymbol{q}]$ have size $3N_c$, and that of $[\boldsymbol{v}^f]$ and $[\boldsymbol{f}]$ is $3N_f$, where N_c is the total number of nodes on the chamber surface, the reticulum and the cone cell ring, and N_f is the total number of nodes on the flagella. The matrix size of $[J^{cc}]$ is $3N_c\times 3N_c$ and $[J^{cf}]$ is $3N_c\times 3N_f$. Considering the boundary condition Eq. (13), the equations can be rewritten as

$$\begin{bmatrix} J^{cc} & J^{cf} \\ J^{fc} & J^{ff} \end{bmatrix}\begin{bmatrix}\boldsymbol{q}\\ \boldsymbol{f}\end{bmatrix} = \begin{bmatrix} 0 \\ \boldsymbol{v}^{fla}\end{bmatrix}. \qquad [16]$$

The dense matrix was solved using the lower-upper (LU) factorization technique.

3. Point force model

A recurring theme in biological fluid dynamics is the representation of flow fields around multiflagellated organisms by suitable singularities in Stokes flow. For example, experimental studies of freely-swimming colonies of the green alga *Volvox carteri*, a spheroid consisting of $\sim 10^3$ biflagellated cells on its surface, have shown a dominant far-field behavior associated with a Stokeslet arising from the density offset between the colony and the surrounding water; a single point force accurately summarizes the effects of a thousand cilia (10). At smaller scales, an accurate representation of the swirling flows near a single biflagellated alga *Chlamydomonas reinhardtii* when it swims in a breaststroke fashion requires three point forces: one for the cell body and one each for the opposing flagella (10, 11).

Returning to the densely-packing choanocyte chambers, it is natural to examine the extent to which the fluid dynamical properties we have found in the numerical studies described above can be represented by the action of one or several point forces. Such a representation would be useful in understanding the input-output characteristics of the chamber, particularly within a coarse-grained model of the the sponge network.

In the original full simulation, there were flagella, the reticulum and the cone cell ring inside a spherical chamber, and the flow was generated as shown in Fig. S3(a). We then integrated the forces acting on these internal structures consolidated them into a set of point forces. Fig. S3(b-d) shows the flow field when the internal structure is divided almost equally into M sections and M point forces are applied at the indicated locations, for the cases (b) $M = 35$, (c) 10 and (d) 1 point force at the sphere center. In all of these coarse-grained models, a flow can be observed from the center of the spherical chamber towards the apopyle.

Fig. S3(a-d) also show the pressure field. Due to the different distribution of forces, the pressure distribution is also altered from one value of M to another, but for all values $M > 1$ there is a similar scale of the high pressure acting at the centre of the chamber. It is only when $M = 1$ that there is a significant reduction in the central pressure. This can be seen in the flow rate of the coarse-grained models relative to that in the fully-resolved numerics, shown in Fig. S3(e). Even for M as small as 10 flux is quantitatively matched within a few percent. But the single point force drastically underestimates the flux as a direct consequence of underestimating the central pressure. We conclude that the effects of flagella around the apopyle, opposed to the directional flow, are indeed crucial to obtain the scale of flux from choanocyte chambers seen in experiment. At the same time, a significantly simplified representation of the chamber is possible.

4. Pressure difference

When the coanocyte chamber faces pressure difference during acting pump, we can consider effects of pumping and background pressure separately in Stokes flow. We investigated the effect of opposing pressure on fluid flow without flagellar motion in addition to flagella driven flow without ambient pressure as shown in Fig. S4. Pressure difference across the chamber is defined as $\Delta P = P_{out} - P_{in}$, where P_{out} is the pressure at outside of the apopyle and P_{in} is the pressure at outside of the prosopyle. Leaky flow rate and pressure at center of the chamber should be linear to ΔP, and can be $C_1 \Delta P$ and $C_2 \Delta P$. High pressure zone is set a little distance away from the apopyle, and we determin P_{out} around the apopyle. Given that P_{in} is set to 0, it follows that $\Delta P = P_{out}$, which in turn implies that $C_1 = Q_p / P_{out}$ and $C_2 = P_c / P_{out}$, where Q_p is the flow rate through the prosopyles and P_c is the pressure at center of the chamber. We calculated C_1 and C_2 for each opening angle θ_a.

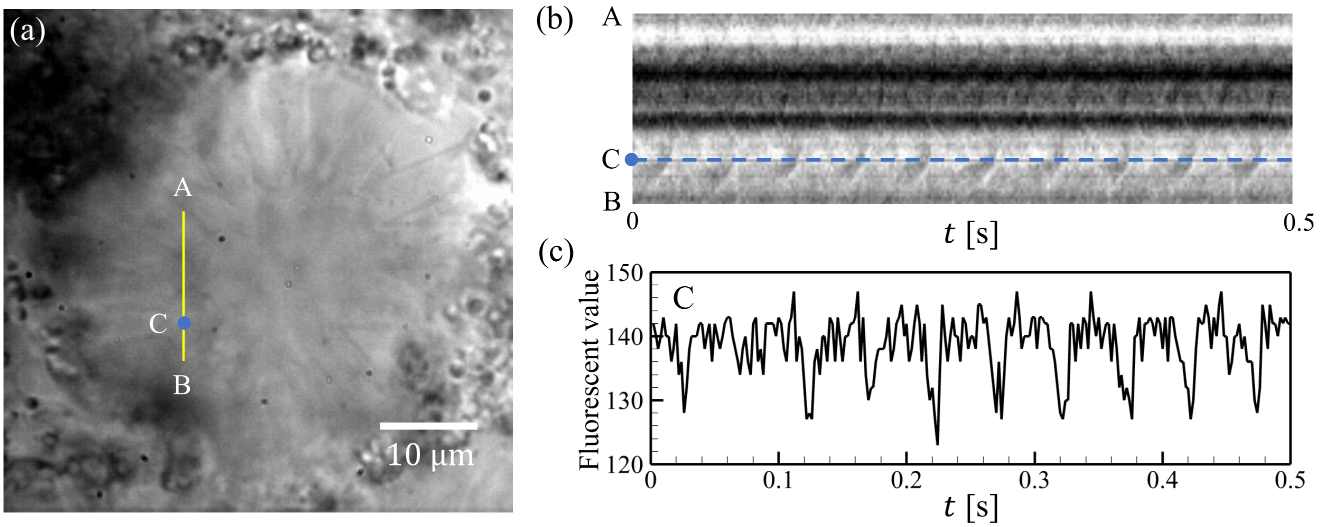

Fig. S1. Flagellar beating in a choanocyte chamber of *E. muelleri*. (a) Microscope image of a choanocyte chamber. (b) Spaciotemporal brightness fluctuation in the choanocyte chamber showing flagellar beating. (c) Slice of data in (b) showing the temporal oscillations in pixel intensity.

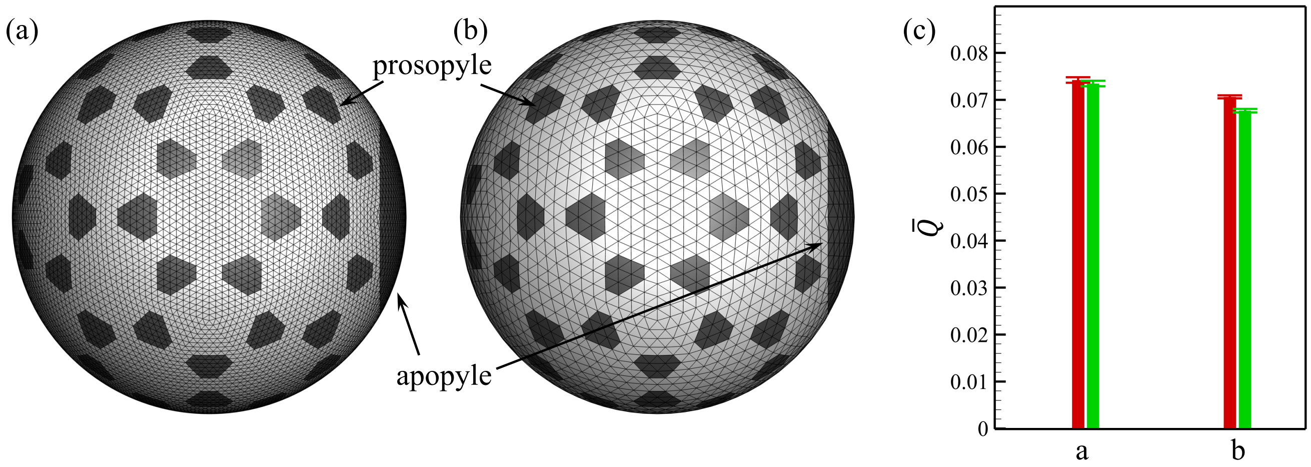

Fig. S2. (a-b) Discretization of the surface of the choanocyte chamber by triangular mesh, and representation of the prosopyles and the apopyle. Mesh (b) is used in the main text, while mesh (a) is used here to check mesh convergence. (c) The flow rate according to fineness of mesh. Red bar is the flow rate through the apopyle, and green bar is total flow rate through the prosopyles.

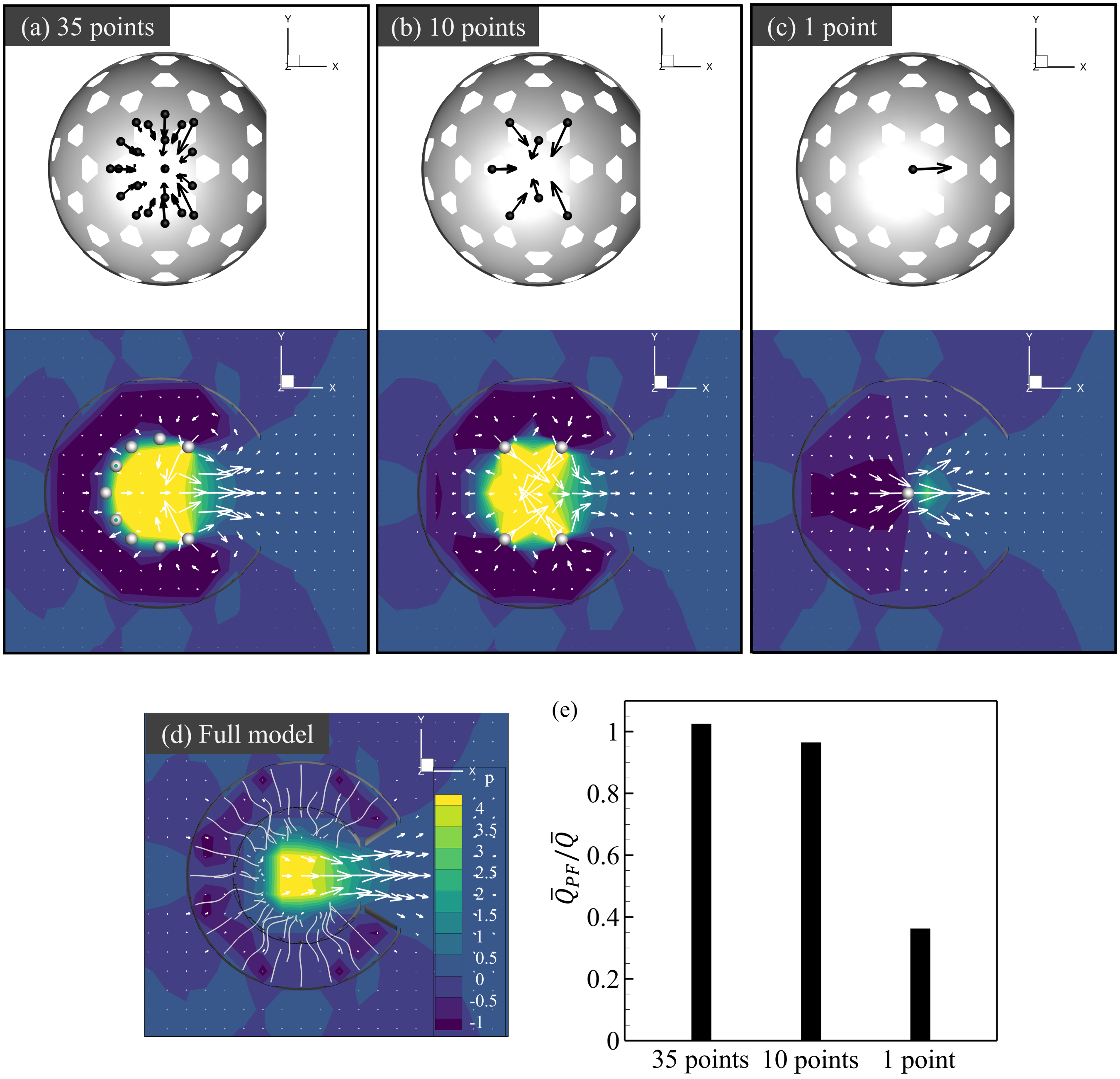

Fig. S3. Flow generated by coarse-grained models of a choanocyte chamber. (a-d) Flow and pressure fields generated by 35 point forces (a), 10 point forces (b), and a single point force (c), the full simulation (d), where dots indicate the positions of the point forces and black arrows indicate the time-averaged forces. (e) Flow rate of the coarse-grained models $\bar{Q}_{PF}$ relative to the flow rate of the full simulation $\bar{Q}$.

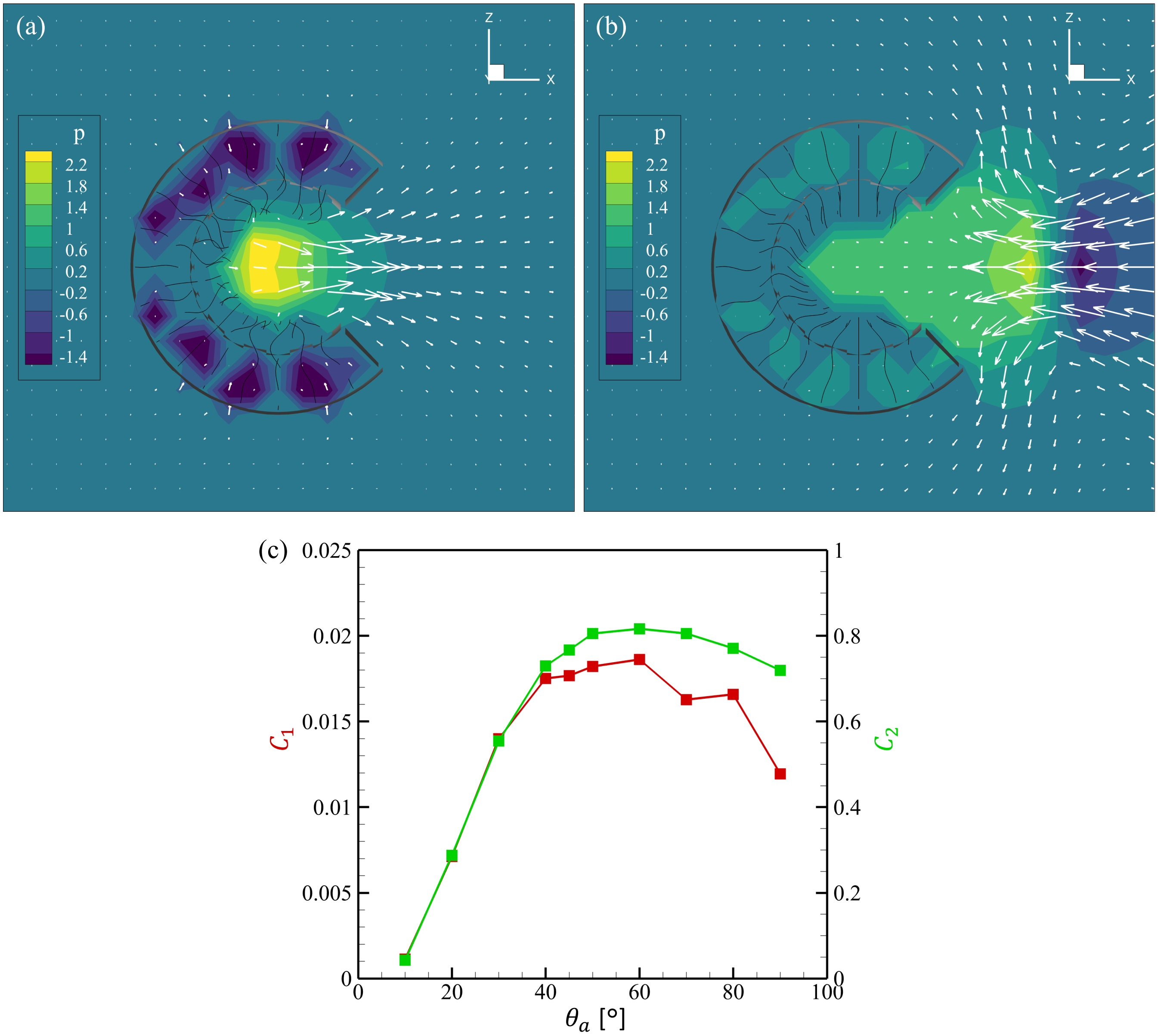

Fig. S4. Flow field and pressure gradient of separated two situation. (a) Flagella drive the flow without ambient pressure. (b) There is only pressure gradient without flagellar motion.

Movie S1. Sectional observation of a choanocyte chamber of *E. muelleri*. This movie speed was decreased 0.25-fold.

References

1. K Kikuchi, T Haga, K Numayama-Tsuruta, H Ueno, T Ishikawa, Effect of fluid viscosity on the cilia-generated flow on a mouse tracheal lumen. *Annals Biomed. Eng.* **45**, 1048–1057 (2017).
2. T Omori, et al., Rheotaxis and migration of an unsteady microswimmer. *J. Fluid Mech.* **930**, A30 (2022).
3. C Pozrikidis, *Boundary integral and singularity methods for linearized viscous flow.* (Cambridge University Press), (1992).
4. HI Andersson, E Celledoni, L Ohm, B Owren, BK Tapley, An integral model based on slender body theory, with applications to curved rigid fibers. *Phys. Fluids* **33**, 041904 (2021).
5. H Ito, T Omori, T Ishikawa, Swimming mediated by ciliary beating: Comparison with a squirmer model. *J. Fluid Mech.* **874**, 774–796 (2019).
6. AK Anna-Karin Tornberg, M Shelley, Simulating the dynamics and interactions of flexible fibers in stokes flows. *J. Fluid Mech.* **196**, 8–40 (2004).
7. E Lac, A Morel, D Barthès-Biesel, Hydrodynamic interaction between two identical capsules in simple shear flow. *J. Fluid Mech.* **573**, 149–169 (2007).
8. M Roper, MJ Dayel, RE Pepper, MA Koehl, Cooperatively generated stresslet flows supply fresh fluid to multicellular choanoflagellate colonies. *Phys. Rev. Lett.* **110**, 228104 (2013).
9. JB Kirkegaard, AO Marron, RE Goldstein, Motility of colonial choanoflagellates and the statistics of aggregate random walkers. *Phys. Rev. Lett.* **116**, 138102 (2013).
10. K Drescher, R Goldstein, N Michel, M Polin, I Tuval, Direct measurement of the flow field around swimming microorganisms. *Phys. Rev. Lett.* **105**, 168101 (2010).
11. T Ishikawa, Fluid dynamics of squirmers and ciliated microorganisms. *Annu. Rev. Fluid Mech.* **56**, 119–145 (2024).
